# The clinical and virological features of two children's coinfections with human adenovirus type 7 and human coronavirus-229E virus

**DOI:** 10.3389/fpubh.2022.1048108

**Published:** 2022-11-15

**Authors:** Shelan Liu, An Zhu, Jinren Pan, Lihong Ying, Wanwan Sun, Hanting Wu, Haiying Zhu, Haiyan Lou, Lan Wang, Shuwen Qin, Zhao Yu, Jian Cai, Yin Chen, Enfu Chen

**Affiliations:** ^1^Department of Infectious Diseases, Zhejiang Provincial Center for Disease Control and Prevention, Hangzhou, China; ^2^Department of Pediatrics, Second People's Hospital of Jinyun County, Lishui, China; ^3^Department of Infectious Diseases, Jinyun District Center for Disease Control and Prevention, Lishui, China; ^4^School of Public Health, Zhejiang Chinese Medical University, Hangzhou, China; ^5^Department of Radiology, First Affiliated Hospital, Zhejiang University School of Medicine, Hangzhou, China; ^6^Department of Geriatrics, First Affiliated Hospital, Zhejiang University School of Medicine, Hangzhou, China; ^7^Department of Microbiology, Zhejiang Provincial Center for Disease Control and Prevention, Hangzhou, China

**Keywords:** HAdV-Ad7, respiratory tract infections, coinfections, case-control study, children, human coronavirus-229E(HCoV-229E)

## Abstract

**Objective:**

Human adenovirus (HAdV) coinfection with other respiratory viruses is common, but adenovirus infection combined with human coronavirus-229E (HCoV-229E) is very rare.

**Study design and setting:**

Clinical manifestations, laboratory examinations, and disease severity were compared between three groups: one coinfected with HAdV-Ad7 and HCoV-229E, one infected only with adenovirus (mono-adenovirus), and one infected only with HCoV-229E (mono-HCoV-229E).

**Results:**

From July to August 2019, there were 24 hospitalized children: two were coinfected with HAdV-Ad7 and HCoV-229E, and 21 were infected with a single adenovirus infection. Finally, one 14-year-old boy presented with a high fever, but tested negative for HAdV-Ad7 and HCoV-229E. Additionally, three adult asymptotic cases with HCoV-229E were screened. No significant difference in age was found in the coinfection and mono-adenovirus groups (11 vs. 8 years, *p* = 0.332). Both groups had the same incubation period (2.5 vs. 3 days, *p* = 0.8302), fever duration (2.5 vs. 2.9 days, *p* = 0.5062), and length of hospital stay (7 vs. 6.76 days, *p* = 0.640). No obvious differences were found in viral loads between the coinfection and mono-adenovirus groups (25.4 vs. 23.7, *p* = 0.570), or in the coinfection and mono-HCoV-229E groups (32.9 vs. 30.06, *p* = 0.067). All cases recovered and were discharged from the hospital.

**Conclusion:**

HAdV-Ad7 and HCoV-229E coinfection in healthy children may not increase the clinical severity or prolong the clinical course. The specific interaction mechanism between the viruses requires further study.

## What is new?

This preliminary study is the first to investigate the differences between groups coinfected with HAdV-Ad7 and HCoV-229E versus a single adenovirus infection. This research for the extremely small sample size found that coinfection with HAdV-Ad7 and HCoV-229E in healthy children may not increase the clinical severity or prolong the clinical course.

## Introduction

Human adenoviruses (HAdVs) are non-enveloped, double-stranded DNA viruses in the *adenoviridae* family (1). Since the first isolation of the adenovirus in 1953, seven species (A–G) have been recognized, including 113 known genotypes or serotypes of HAdV (2–6). More than 60 genotypes are known to cause human infection (7). Adenoviruses can cause illness in people of all ages at any time of the year (8). Most children have had at least one adenovirus infection by age 10 (9). Globally, 5–7% of respiratory tract infections in pediatric patients are ascribed to HAdV (10, 11).

Human coronaviruses (HCoVs) are enveloped, single-stranded RNA viruses (12, 13).

HCoV-229E is one of seven HCoVs: HCoV-229E, HCoV-NL63, HCoV-OC43, HCoV-HKU1, severe acute respiratory syndrome coronavirus (SARS-CoV), Middle East respiratory syndrome coronavirus (MERS-CoV), and severe acute respiratory syndrome coronavirus 2 (SARS-CoV-2) (14–16). HCoV-229E usually causes mild to moderate upper-respiratory tract illness, similar to the common cold (17). People generally become infected with HCoV-229E in the fall and winter, but infection can occur at any time of the year (17).

Human adenovirus (HAdV) is commonly associated with acute respiratory illnesses (ARI) in children and is also frequently co-detected with other viral pathogens (18). HAdV co-detection with other respiratory viruses was associated with greater disease severity among children with ARI compared to HAdV detection alone (18). However, coinfection with HCoV is very rare and unusual. For example, national data from China indicate a rate of 7.2% for coinfection in hospitalized acute lower respiratory infections (ALRIs), with the most common pairs being adenovirus + respiratory syncytial virus (9.71%), followed by human parainfluenza viruses + adenovirus (3.86%); and influenza + adenovirus (3.45%) (19). Pathogen coinfection occurred only two times between adenovirus and hCoV (0.94%) (19). Additional research from Beijing indicated that, among 90 HAdV-positive children, 61.11% (55/90) were coinfected with other respiratory viruses, the most common of which were human respiratory syncytial virus (34.5%) and human rhinovirus (10.9%). The fewest cases of coinfection occurred with adenovirus and HCoV (only 1.8%) (20). To date, there are no documented adenovirus coinfections with HCoV-229E, so the clinical outcome of this pair of coinfections remains unknown.

An adenovirus outbreak was recognized in 2019 in Lihui city, Zhejiang Province, China. This outbreak involved 97 cases, including 24 admitted cases in the pediatric ward, with two confirmed coinfections of adenovirus and HCoV-229E. In order to compare clinical presentation and outcomes, and virological features in young children with HAdV detected alone vs co-detected with HCoV-229E, we explored a rare phenomenon in this study.

## Materials and methods

### Study design and participants

China's national surveillance system for influenza-like illness (ILI), severe acute respiratory illness, and pneumonia of unexplained origin indicated an outbreak of 97 adenovirus infections during July and August of 2019. Twenty-four children were hospitalized in the pediatric ward of the Second Hospital of JinYun. Two cases were confirmed to be HCoV-229E and adenovirus coinfections, and these children were admitted to different rooms (Rooms 830, 845) in the same pediatric ward. Twenty-one cases were identified as mono-adenovirus. One case involved a 14-year boy with a high fever who tested negative for Ad7 and HCoV-229E; this case was not included in our research.

Through extensive symptom surveillance, we found an additional three people (the parents of one of the coinfections and one doctor) who were confirmed as HCoV-229E positive. HAdV patients coinfected with HCoV-229E were categorized into the research group. For each patient in the research group, we matched three cases with a single HCoV-229E infection and 21 with a single adenovirus infection as controls.

The diagnosis of adenovirus and HCoV-229E followed the Protocol for Adenovirus Pneumonia Diagnosis and Treatment issued by the National Health Commission of the People's Republic of China in 2018.

### Clinical information collection

The patients' clinical manifestations, laboratory examinations, imaging characteristics, disease severity, and clinical progress were collected from electronic medical records in the Second Hospital of JinYun's hospital information system (HIS). Radiologic abnormalities were determined according to descriptions in the clinical charts. All variables were compared between the three groups, including coinfections with adenovirus, mono-adenovirus, and HCoV-229E respectively.

Three methods were used to evaluate the severity and clinical progress of the disease. First, according to the national guidelines for pediatric CAP (community-acquired pneumonia) in China, we divided cases of pneumonia into mild and severe (10). Second, the extrapulmonary manifestations involved in our study included kidney manifestations, myocardial damage, liver damage, and coagulation function. Third, we observed the number of days with fever, the highest degree of fever, and the median days from illness onset to discharge.

### Laboratory testing

We collected specimens from the upper respiratory tract using pharyngeal swabs. All of the patients were laboratory confirmed using real-time reverse transcriptase polymerase chain reaction (RT-PCR) or PCR assays. Laboratory confirmation was also performed on other common respiratory pathogens, including influenza-A virus (H1N1, H3N2), influenza B virus, respiratory syncytial virus (RSV), parainfluenza virus, adenovirus, SARS-associated coronavirus (SARS-CoV), and human coronavirus HCoV-229E. Any coinfections were included from this study. Genetic sequences of viruses were obtained directly from positive clinical specimens or from virus isolates using an MiSeq desktop sequencer (Illumina, Inc., San Diego, CA, USA), as described (21).

### Statistical analysis

Quantitative measurements were presented as medians; qualitative measurements were presented as counts and percentages. Abnormally high or low levels of laboratory findings were defined using age-specific or otherwise universal reference ranges. The differences between groups were analyzed using the Wilcoxon signed-ranks test (for continuous data) or McNemar's chi-square test (for binary data), with a *p*-value of less than 0.05 considered statistically significant. All analyses were conducted with R (version 3.4.6; The R Foundation for Statistical Computing, Vienna, Austria).

## Results

### Demographic characteristics and exposure history

We collected a total of two cases of coinfection with adenovirus and HCoV-229E, three cases with single HCoV-229E infection, and 21 cases with single adenovirus infection. None of the patients had underlying diseases. Patients' median ages were 11, 38, and eight years in the coinfection group with adenovirus and HCoV-229E, the mono-infection group with HCoV-229E, and the mono-infection group with adenovirus, respectively (*p* = 0.332) (Table 1). The male to female ratio was 0:2 in the coinfection group, 1:2 in the mono-infection with HCoV-229E group, and 2.5:1 in the mono-infection with adenovirus group (*p* = 0.111) (Table 1). All of the children with coinfections and adenovirus mono-infection had been to a swimming pool and had been exposed to someone with a fever. However, the three individuals with mono-infections of HCoV-229E had provided bedside and medical care for the two children with coinfections, as shown in Table 1.

**Table 1:** Comparison of the characteristics of two children coinfected with HCoV-229E and adenovirus admitted to different rooms of the same pediatric ward and mono-infections with HCoV-229E and Adenovirus in Lishui, Zhejiang Province, July–August 2019.

**Characteristic**	**Coinfection withHCoV-229E and adenovirus (*****n*** = **2)**		**Mono-infection with HCoV-229E (*****n*** = **3)**			**Mono-infection with adenovirus (*n* = 21)**	***p*-value**
	**Co-case 1**	**Co-case 2**	**Case 1**	**Case 2**	**Case 3**		
**Demographics**							
Age (in years)	13	9	54	59	43	8 (2–14)	0.332
Gender	Female	Female	Female	Male	Female	Male/female = 2.5:1.0	0.111
Occupation	Student	Student	Farmer	Farmer	Doctor	Preschool children or students	/
**Basic situation**							
History of alcohol use	No	No	No	No	No	No	/
History of smoking	No	No	No	Yes	No	No	/
Underlying conditions	No	No	No	No	No	No	/
**Exposure history**							
Visit to the swimming center	Yes	Yes	No	No	No	100%	/
Exposure to a febrile person	Yes	Yes	Yes	Yes	Yes	100%	/
Provided bedside care	No	No	Yes	Yes	No	No	/
Medical care services	No	No	No	No	Yes	No	/
**Laboratory results**							
Specimen collection date	August 2	August 2	August 2	August 2	August 2	August 2	/
Specimen collection type	Throat swab	Throat swab	Throat swab	Throat swab	Throat swab	Throat swab	/
Diagnostic method	rRT-PCR and sequencing	rRT-PCR and sequencing	rRT-PCR and sequencing	rRT-PCR and sequencing	rRT-PCR and sequencing	rRT-PCR and sequencing	/
Date of confirmation	August 5	August 5	August 7	August 7	August 7	August 5	/
viral load (ct value)	Adenovirus (28.8)	Adenovirus (22)	Adenovirus (Negative)	Adenovirus (Negative)	Adenovirus (Negative)	Adenovirus (Average: 23.78)	0.570
	HCoV-229E (32.5)	HCoV-229E (33.3)	HCoV-229E (28.4)	HCoV-229E (30.5)	HCoV-229E (31.3)	HCoV-229E (Negative)	0.067
**Clinical features**							
Max temperature (°C)	40.2	39.8	/	/	/	39.3	0.1431
Fever duration (days)	3	2	/	/	/	2.90	0.5062
Exposure to onset (days)	2	3	No symptoms	No symptoms	No symptoms	3 (0–7)	0.8302
Onset to admission (days)	2	1	/	/	/	2.9 (0–6)	0.332
Hospital stays (days)	6	8	/	/	/	6.76 (3–13)	0.640
Days from onset to be discharged (days)	8	9	/	/	/	9.67 (3–17)	0.285

*CT*, cycle threshold. In this table, all of the statistical analyses were performed using the statistical package for the social sciences (SPSS; version 26.0). Categorical variables were compared using Fisher's exact test. The Mann–Whitney test was used to compare continuous variables. All statistical tests were two-sided, and a *p*-value of 0.05 was considered statistically significant. The symbol / indicates that data are not available.

*P*-value notes the comparison of the coinfection group and the mono-adenovirus group.

### Comparison of clinical characteristics

#### Coinfection-case 1

**Figure 1:**
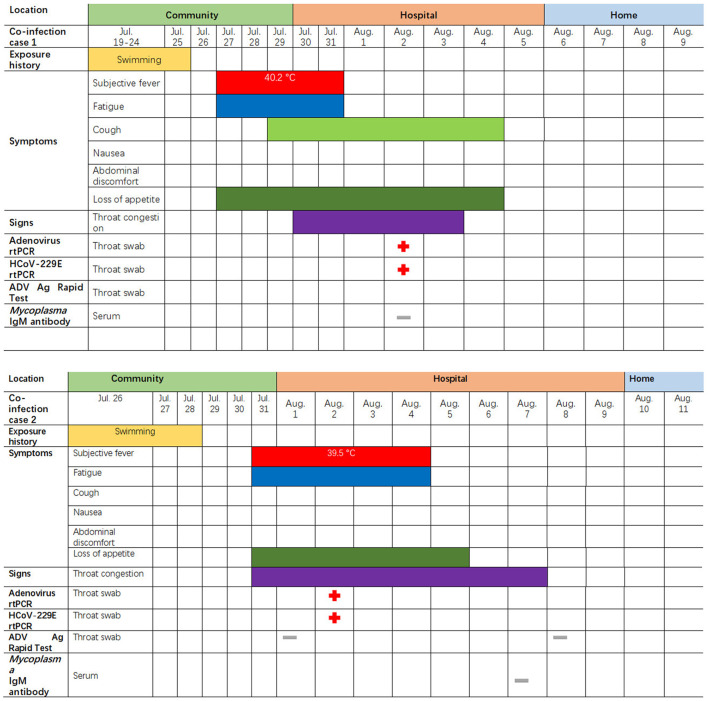
Symptoms and results of rtPCR testing and antigen rapid test for the two children coinfected with HCoV-229E and adenovirus; they were admitted to different rooms in the same pediatric ward in Lishui, Zhejiang Province, July–August 2019. rtPCR = real-time PCR. ADV = adenovirus. The red cross indicates that the pathogens (adenovirus or 229E) were detected by PCR or rapid antigen detection. The gray – indicates an HCoV-229E negative test.

**Figure 2:**
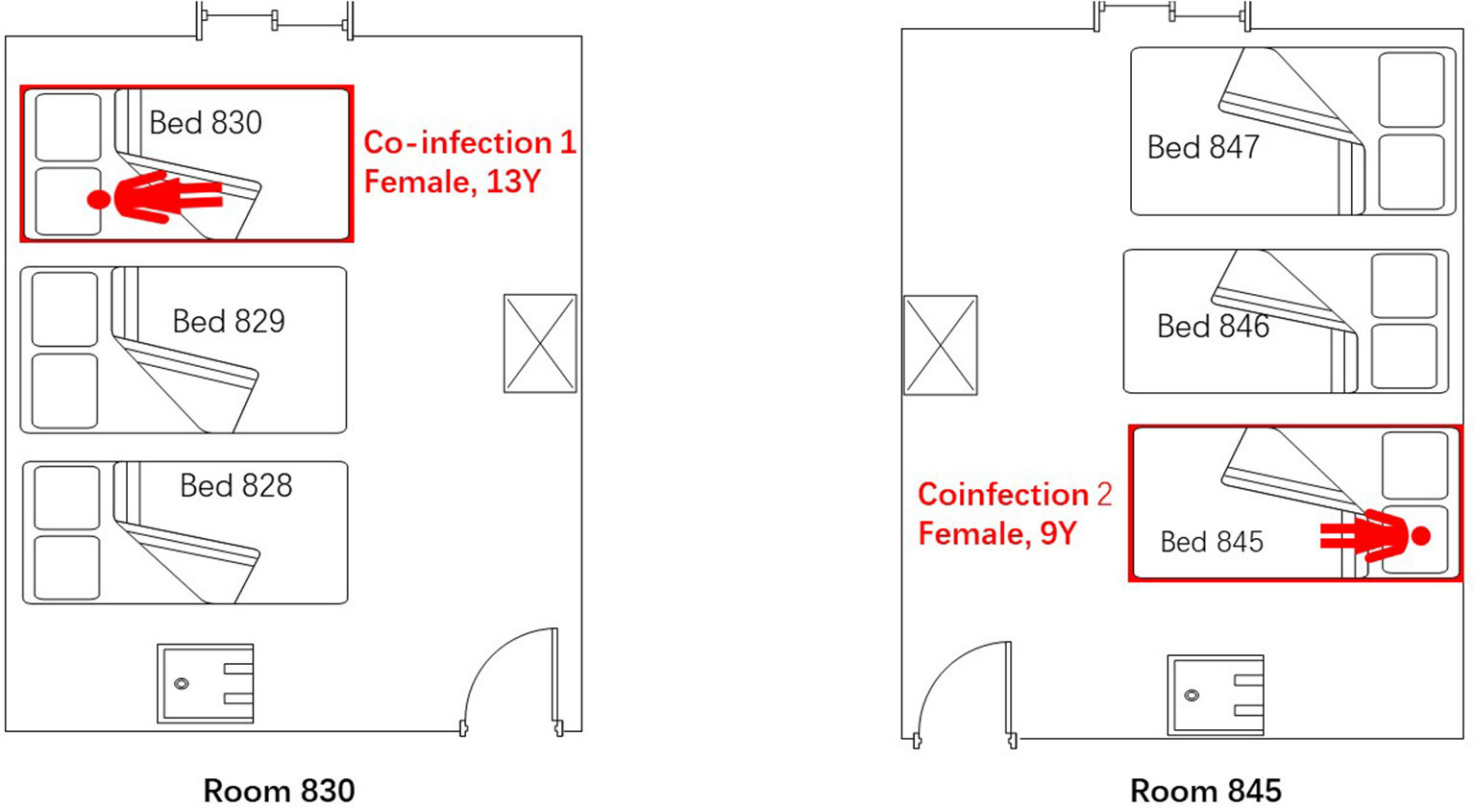
Spatial distribution of the two admitted children who were coinfected with adenovirus and HCoV-229E in the pediatric ward of Lishui, Zhejiang Province, July–August 2019.

**Figure 3:**
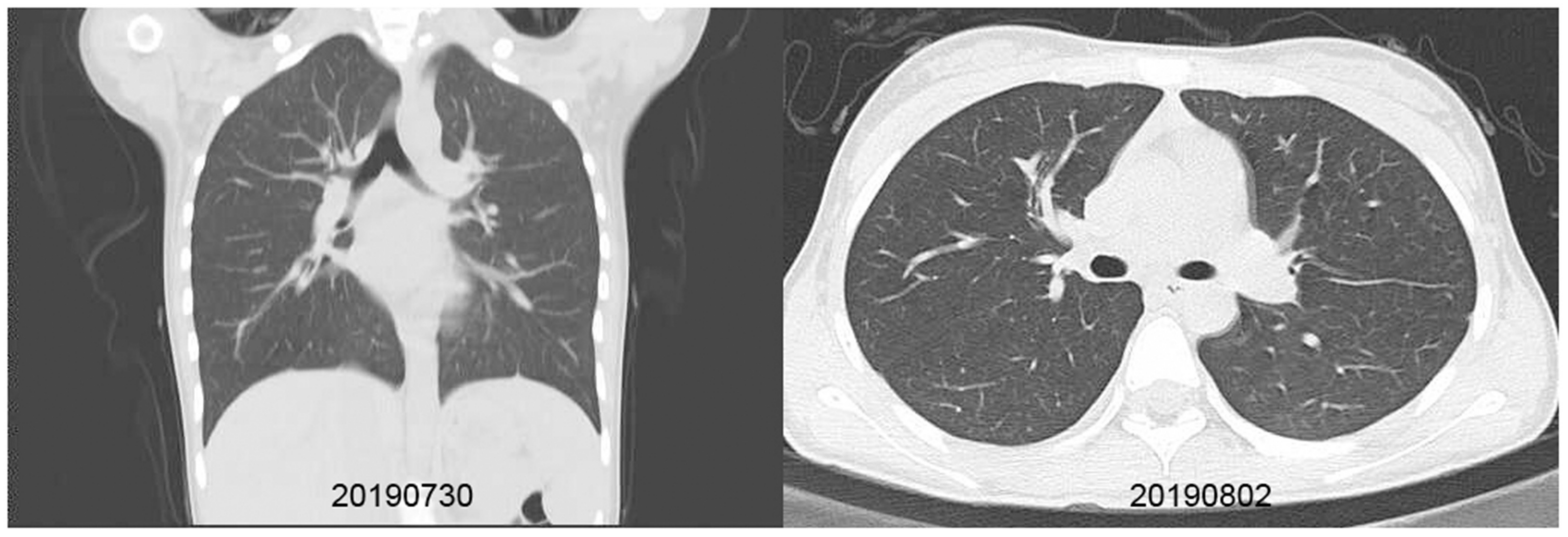
Chest X-ray and CT scan taken for a 13-year-old girl with HCoV-229E and adenovirus coinfection-induced fever on July 27, 2019. Chest CT scan and chest X-ray indicated slight thickening of the wall of the anterior and posterior segments of the right upper lobe, a slightly narrower lumen, and slight inflammation of the lower right lung. The bilateral hilum was normal, and no pleural effusion was observed. No obvious swollen lymph node shadow was found in the mediastinum. **(A)** Left, Chest X-ray taken on July 30, 2019. **(B)** Right, Chest CT scan taken on August 2, 2019.

The first coinfection patient was a 13-year-old girl (a student from Jinyun) who had no underlying diseases. She had taken swimming classes at a swimming center on July 25, 2019, where an adenovirus outbreak was identified, as seen in Figure 1. She was admitted to room 830 of the pediatric ward on July 30 (see Figure 2), presenting with a dry cough, a continual fever for two days with an initial temperature of 38.6°C and a high temperature of 40.2°C. She had no gastrointestinal symptoms. On physical examination, she had a body mass index of 23.5 kg/m^2^, and her vital signs were as follows: a heart rate of 120 beats per minute, 28 breaths per minute, an oxygen saturation of 95% at ambiance, and pharyngeal congestion oozing. Further examination revealed a decreased neutrophil absolute count and percentage, but normal liver, heart, and kidney functions and C-reactive protein (CRP) levels, as seen in Table 2. Chest radiography, performed on August 2 following disease onset, indicated slight thickening of the wall of the anterior and posterior segments of the right upper lobe, with the lumen slightly narrowed and the lower right lung slightly inflamed (Figure 3). Symptomatic treatment was started on the day of illness onset, without any oxygen or glucocorticoid therapy. The fever and cough resolved by August 1 and August 4, respectively (Figure 1). A throat swab sample collected on August 2 was positive for adenovirus (ct value: 28.8) and HCoV-229E (ct value: 32.5), as seen in Table 1. On August 5, the patient was discharged and recovered completely (Table 1). The period from illness onset to discharge was eight days (see Table 1 and Figure 1).

**Table 2:** Hematological and blood biochemical measurements of two children coinfected with HCoV-229E and adenovirus admitted to different rooms of the same pediatric ward and mono-infections with adenovirus in Lishui, Zhejiang Province, July–August 2019.

**Variables**	**Coinfection-case 1**	**Coinfection-case 2**	**Mono-infections with adenovirus (*n* = 21)**	**Normal range**	***p*-value**
**Blood routine**					
WBC (×10^9^ per L)	2.1	4.7	5.66	4.0–10.0	0.347
Lymphocyte absolute count (×10^9^ per L)	1.22	1.02	2.61	0.8–4	0.06
Neutrophil absolute count (×10^9^ per L)	0.63	3.17	2.11	2–7.7	0.764
Lymphocyte percentage (%)	59.54	21.90	50.57	20–40	0.648
Neutrophil percentage (%)	30.74	68.00	42.10	50–70	0.876
Platelet count (×10^9^ per L)	210	201	234.91	100–300	0.465
Hemoglobin (g/L)	115	127	119.32	110–150	0.917
**Blood biochemistry**					
Albumin (g/L)	39.7	36.5	39.48	35.0–55.0	0.958
ALT (U/L)	7	13	15.64	0–40	0.295
AST (U/L)	24	28	26.77	0–40	0.793
Urea (mmol/l)	3.60	3.14	3.84	2.50–7.20	0.497
Creatinine(μmol/L)	50	38	42.41	44–132	0.754
C reactive protein (mg/dl)	3.0	0.5	5.61	0–10	0.825
Lactate dehydrogenase (UI/liter)	186	229	240.82	65–220	0.230

*AST*, alanine aminotransferase; *ALT*, aspartate aminotransferase.

#### Coinfection-case 2

The second co-case patient was a healthy nine-year-old girl who had visited the same swimming center as the first patient on July 28, 2019. She was admitted to room 845 of the pediatric ward on August 1 because of a history of high fever, 38.6°C for one day (Figures 1, 2). The fever continued for four days, reaching a high of 39.5°C. Upon physical examination, the patient had pharyngeal congestion oozing. The lab test indicated that the patient had a normal blood count, liver function, and kidney function, but slightly increased lactate dehydrogenase (Table 2). A throat swab sample collected on August 2 was positive for adenovirus (ct value: 22) and HCoV-229E (ct value: 33.3), as shown in Table 1. Symptomatic treatment and support therapy was started, without any glucocorticoid therapy. The patient was discharged eight days after admission (see Table 1 and Figure 1).

#### Mono-infection with HCoV-229E

The parents of coinfection-case 1 (mother: 54 years old, father: 59 years old, both farmers) undertook bedside care and remained asymptomatic (Table 1). A 43-year-old female doctor without any symptoms was in charge of the two co-cases. Three throat samples collected on August 6 showed that the parents (ct values: 30.5, 28.4, respectively) and doctor had HCoV-229E (ct value: 31.3) (Table 1).

#### Mono-infection with adenovirus

All patients infected with a single adenovirus showed mild illness, with no serious illness found (Table 3). The most common clinical symptoms or signs were fever (100%, 22/22), fatigue (40.91%, 9/22), sore throat (40.91%, 9/22), and vomiting (36.36%, 8/22) (Supplementary Figure 1). The highest fever temperature (40 vs. 39.3°C, *p* = 0.1431) and fever duration (2.5 vs. 2.9 days, *p* = 0.5062) were the same between the coinfection and mono-infection with adenovirus groups (Table 1). We compared the clinical progress among the 21 mono-adenovirus infections with the HAdV-7 group and two coinfections with HAdV-7. Between the coinfection group and mono-HAdV-7 group, the results indicated no differences in average days from exposure to onset (2.5 vs. 3 days, *p* = 0.8302), onset to admission (1.50 vs. 2.90 days, *p* = 0.332), hospital stay (7 vs. 6.76 days, *p* = 0.640), or onset to discharge (8.50 vs. 9.67 days, *p* = 0.285) (see Table 1).

**Table 3:** Treatment and disease severity of two children coinfected with HCoV-229E and adenovirus admitted to different rooms of the same pediatric ward and mono-infections with adenovirus in Lishui, Zhejiang Province, July–August 2019.

**Variables**	**Co-case 1**	**Co-case 2**	**Mono-infections with adenovirus (*n* = 21)**	***p*-value**
Mild CAP	Yes	No	One case	Not available
Severe CAP	No	No	No	Not available
Extremely severe pneumonia	No	No	No	Not available
PICU admission	No	No	No	Not available
Immune global protein	No	No	No	Not available
Glucocorticoids therapy	No	No	One two-year-old child	Not available
NCPAP	No	No	No	Not available
Invasive mechanical ventilation	No	No	No	Not available
Oxygen therapy	No	No	No	Not available
Clinical outcome	Survival	Survival	Survival	Not available

*CAP*, community-acquired pneumonia; *NCPAP*, nasal continuous positive airway pressure; *PICU*, the pediatric intensive care unit.

#### One coinfection with adenovirus mycoplasma pneumoniae

**Figure 4:**
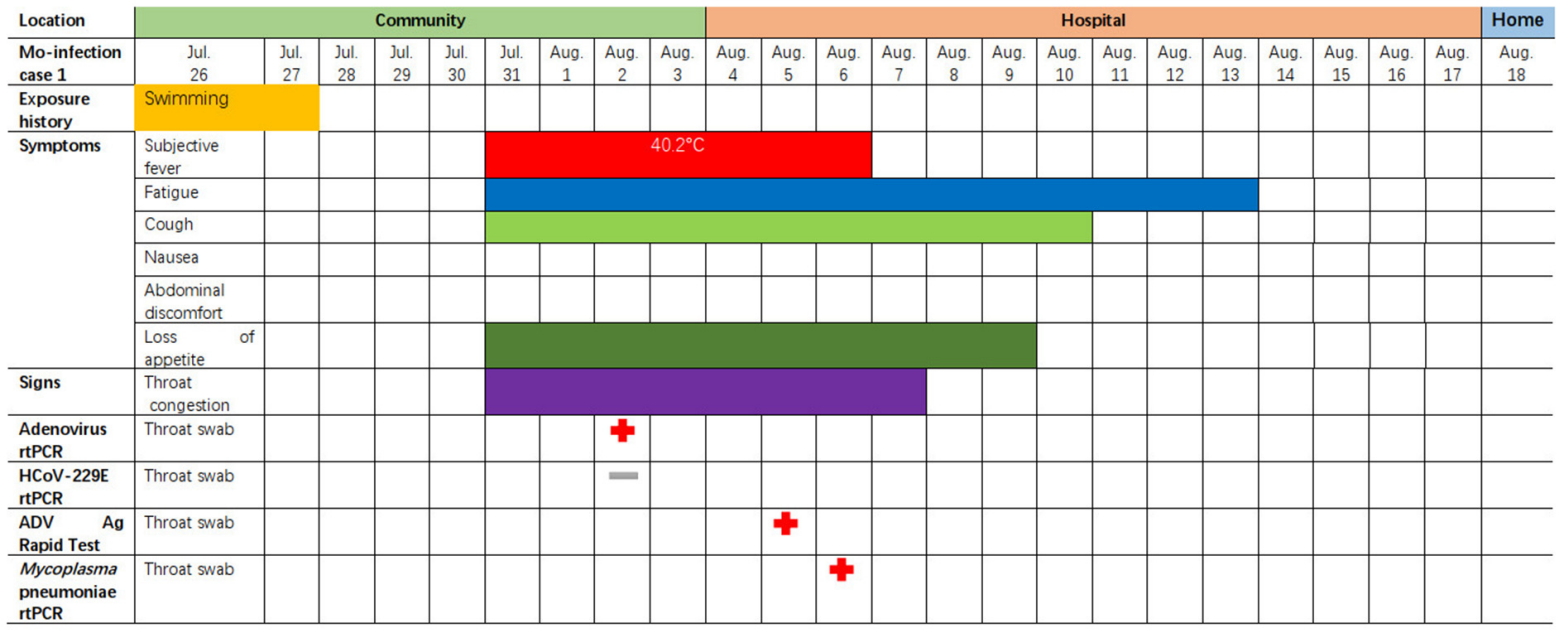
Symptoms and results of rtPCR testing and antigen rapid test for a two-year-old child coinfected with adenovirus and *single M. pneumoniae* who was admitted to the pediatric ward in Lishui, Zhejiang Province, August 4–17, 2019. rtPCR = real-time PCR. ADV = adenovirus. The red cross indicates that the pathogen (adenovirus or single *M. pneumoniae*) was detected by PCR or rapid antigen detection. The gray—indicates an HCoV-229E negative result.

**Figure 5:**
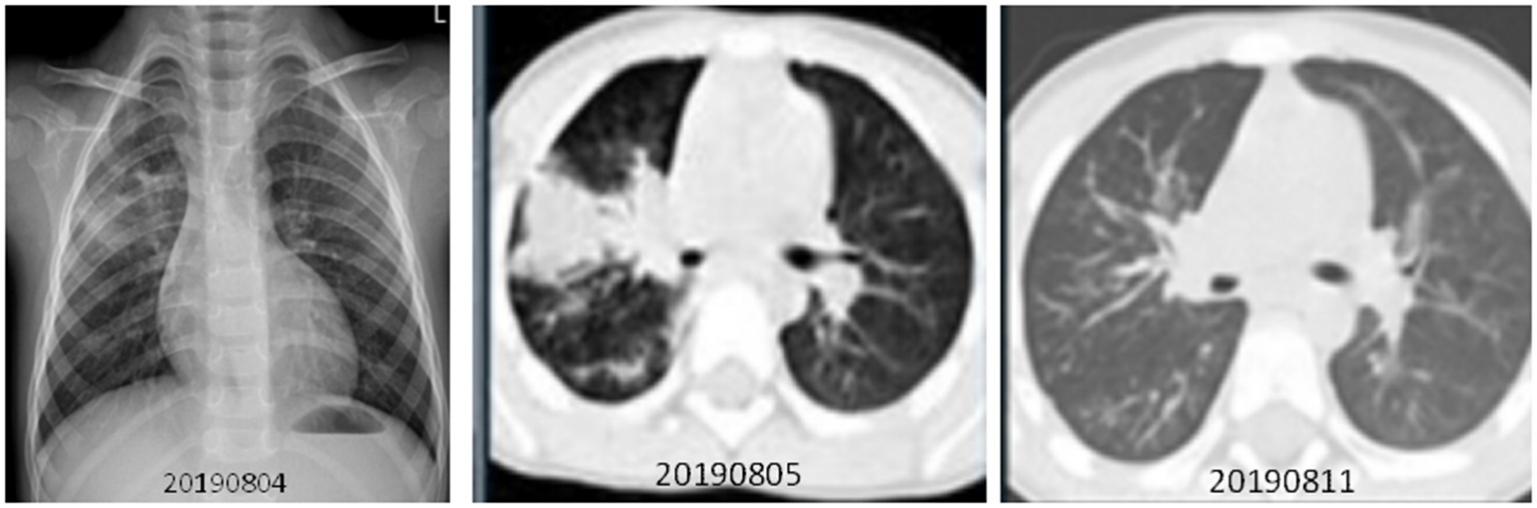
Chest X-ray and CT scan were taken for a two-year-old boy with adenovirus and *mycoplasma pneumoniae* coinfection-induced fever on July 31, 2019. (1) **(A)** Left, Chest X-ray taken on August 4, 2019. Exudation and small patchy shadows in the upper lobe of the right lung. The right hilum shadow was slightly enlarged, and the left hilum was normal. No bilateral pleural effusion was observed. (2) **(B)** Middle, Chest CT plain scan was taken on August 5, 2019. Incomplete, segmental lung consolidation and exudation lesions were observed. Signs of air bronchogram were seen in the consolidation tissue, accompanied by segmental consolidation in the dorsal segment of the right lower lobe. The right hilar shadow was enlarged, and the left hilar was normal. There was no bilateral pleural effusion and no obvious enlarged lymph nodes in the mediastinum. **(C)** Right, Chest CT plain scan taken on August 11. The upper lobe of the right lung was redilated, showing upper, middle, and lower lobe bronchial wall thickening in the right lung as well as a weak patchy shadow distributed along the airway. There was no thickening of the lobular septum or pleura, and no pleural effusion was observed. The hilar had a normal morphology, and there was no significantly enlarged lymph node shadow in the mediastinum. Compared with the CT scans from the week prior, the right lung lesion had been significantly absorbed.

The case patient was a healthy two-year-old boy who had visited a JinYun swimming center on July 27, 2019. On July 31, he developed a constant high fever of 40.2°C and a cough, leading to admission to room 840 of the pediatric ward on August 4 (Figure 4). The lab test indicated that the patient had an elevated white blood cell count, an increased lymphocyte percentage, a decreased neutrophil percentage, and a low level of hemoglobin (113 g/L). A chest X-ray was taken on August 4, 2019, depicting exudation and small patchy shadows in the upper lobe of the right lung. In addition, the right hilum shadow was slightly enlarged (Figure 5A). A chest CT (Computed Tomography) scan on August 5 showed signs of an air bronchogram in the consolidation tissue, accompanied by segmental consolidation in the dorsal segment of the right lower lobe (see Figure 5B). On August 5, the mycoplasma DNA loads by rt-PCR were 3.60 × 10^4^ copies/ml (normal level: <400) (Figure 4). A throat swab sample collected on August 2 was positive for adenovirus (ct value: 21.4) (Figure 4). A chest CT plain scan taken on August 11 indicated that the upper lobe of the right lung was redilated (Figure 5C). The patient was discharged on August 17 after the use of glucocorticoids (Figure 4 and Table 3). None of the above patients were treated with invasive mechanical ventilation, transferred to the PICU (Pediatric Intensive Care Unit), or given oxygen therapy (Table 3).

#### Comparison of viral loads and genetic identity

The average viral ct was 25.4 and 23.7 in the coinfection and single adenovirus groups (*p* = 0.570), while the average viral ct was 32.9 and 30.06 in the coinfection and single HCoV-229E groups (*p* = 0.067), respectively (Table 1). The difference in viral loads was not statistically significant between the two groups (Table 1).

Regarding the alignment of the penton, fiber, and hexon of the adenovirus isolated from the coinfection group (*n* = 2) and single adenovirus group (*n* = 21), these adenovirus sequences shared 99.5 and 100% of the nucleic acid sequence identity. The HCoV-229E S1 gene segments for the coinfection group (*n* = 2) and single HCoV-229E group (*n* = 3) were 97.5–99.9% identical.

## Discussion

We report on the rare phenomenon of HAdV-Ad-7 and adenoviral HCoV-229E coinfection in children. Compared with HAdV-Ad-7 mono-infection, there was no increase in clinical severity for children coinfected with HCoV-229E. There were also no differences in terms of incubation, fever duration, high temperature, length of hospital stays, or viral load between the two groups. The three adults infected with HCoV-229E did not develop any symptoms. No patients with coinfections or mono-infections of HAdV-Ad-7 or HCoV-229E had severe complications; they fully recovered after early and adequate supportive and symptomatic treatment.

Several studies have reported on HAdV-Ad-7 coinfections with other pathogens (19). One study indicated that 4% of pediatric pneumonia admissions were associated with endemic HCoVs, with a high proportion of cases co-occurring with another respiratory virus (22, 23). However, coinfection with HCoV-229E and adenovirus has not been documented in the literature. In this study, our joint investigation team continuously sampled the outbreak cases (symptomatic and asymptomatic patients) and used a more sensitive method (Multiplex PCR method for simultaneous tests of 14 different pathogens). Finally, two co-infections were isolated from the 24 admitted cases of human adenovirus genotype 7, accounting for 8.33% (2/24) of the total number of cases. This number was far lower than adenovirus coinfected with other pathogens, such as respiratory syncytial virus (19/37, 34.5%) and influenza virus (6/37, 10.9%] (20). The exact reasons for the coinfections remain unclear, but several factors may have played a role. Most importantly, the children infected with adenovirus were weakened and susceptible to other pathogens after the persistent high fever. Secondly, there are no vaccines against adenovirus or HCoV-229E virus in China. Meanwhile, HCoV-229E has a low prevalence in this area. As a result, most of population has no immunity to these two viruses (24). Thirdly, adenoviruses are non-enveloped viruses that are unusually resistant to physical and chemical agents, which gives them prolonged survival capacity in various environments (25). The incubation period for mono- or coinfection was 2–3 days, which aligns with previous estimates of adenovirus incubation periods (26). However, mono-adenovirus infection, mono-HCoV-229E infection, and coinfection with HAdV-Ad-7 and HCoV-229E involve non-specific signs and symptoms. This lack of specific symptoms contributes to delayed diagnosis and treatment (27–29).

Furthermore, the present study showed that both groups had similar clinical signs and laboratory test results, such as white blood cell (WBC), AST and ALT, lactate dehydrogenase (LDH), and C-reaction protein (CRP) counts. Therefore, it is difficult to distinguish whether or not patients have a mono-infection with adenovirus or coinfection with two viruses based on clinical signs and routine blood tests. Although coinfection is not common, in cases where the disease is not explained by a single pathogen, additional studies, such as a nested PCR for multi-respiratory panel pathogens, are required to detect potentially treatable pathogens, such as COVID-19 mycoplasma, influenza virus, and 229E (30, 31).

Fever is a manifestation of the body's resistance to inflammation, often used to judge the progress or outcomes of a disease (32). In this study, both groups developed acute illness, characterized by a constant high fever; they also had similar fever durations (3–5 days), which was not consistent with previous reports (33). Chen et al., for instance, indicated that 6/103 of the children in their study had HAdV coinfected with *mycoplasma pneumoniae*, and the proportion of fever duration >10 days (40.8%) in the mixed infection group was significantly higher than in the mono-infection group (24.5%, *p* = 0.014) (34). The present study found no significant difference in patient age and sex between the mono- and coinfection groups. The median duration of hospital stay in the two groups was generally shorter than eight days, which aligns with information on single adenovirus infections reported from Beijing (20). This limited data suggest that coinfection with two viruses does not prolong the clearance time of the pathogen, aggravate the host immune response, lead to organ damage, or show more internal and exogenous pyrogen.

Regarding the severity of disease, some research has indicated that HAdV has strong virulence that can cause high mortality in children (32). HAdV-7 in particular may cause severe infection (35, 36). One study indicates that HAdV coinfection with another pathogen aggravates the severity of pneumonia in children (10). Studying the risk factors of severe atypical pneumonia using multivariate logistic regression analysis, Huong et al. found that coinfection with a respiratory virus was a risk factor for severe atypical community-acquired pneumonia in children (OR = 4.36, *p* = 0.008) (37). Previous studies indicated that HAdV coinfection with another pathogen aggravates the severity of pneumonia in children (10). For example, Juan et al. reported that adenovirus and SARS-CoV-2 mixed infections are associated with adverse clinical outcomes, such as shock, lymphopenia, and thrombocytopenia. Patients with these mixed infections are also more likely to require ventilatory support and admission to intensive care units (38). However, these studies are not consistent with our findings, indicating that both mono- and coinfections typically resulted in mild upper respiratory tract infections. Furthermore, the cases in this study also did not show any liver damage, which differed from other studies that found the adenovirus 41 genotype to lead to hepatitis of unknown origin (39–44). Only one 13-year-old girl, coinfected with adenovirus and HCoV-229E, and one two-year-old boy with adenovirus and mycoplasma, developed mild pneumonia, without any severe complications. Both patients were cured and discharged after the use of symptomatic support therapy and glucocorticoids. The severity of HAdV infection is affected by many factors, including the patient‘s age, immune status, diagnosis, viral load, and socioeconomic status (20). In this study, all cases presented mild illness regardless of whether they had a single infection (adenovirus or HCoV-229E) or coinfection with two viruses. Several factors may have contributed to this outcome. First, all coinfections and mono-infections were in young children who had no underlying diseases or need for regular medicine administration. By contrast, adenovirus has been recognized as a cause of severe illness among immunocompromised children (45). Second, diagnosis and therapy was provided early and in a timely fashion for these cases. Third, the viral loads in the two groups were at the low to middle level, without any mutations associated with viral reproduction. However, the acute mechanism of severity following HAdV-7 and HCoV-229E co-infections should be further investigated in the future.

In summary, this study explored the unusual phenomenon of HAdV-7 and HCoV-229E coinfection in healthy children. Compared with single adenovirus infections, coinfection with HAdV-Ad7 and HCoV-229E virus did not contribute to disease severity, a finding that may be attributed to the children's good underlying health and low viral loads. Other factors in these positive outcomes include early identification of a potential adenovirus coinfection and successful treatment. However, larger and better-designed prospective analytical studies are required to examine further risk factors as well as the interaction between adenoviruses and other respiratory pathogen coinfections.

This study has some shortcomings. First, as a retrospective study, data are inevitably missing; for example, the serum antibody counts against the adenovirus and HAdV or HCoV-229E and the systemic inflammatory cytokines were not available for the mono-infections or coinfections. Second, the number of ADV+ HAdV-229E coinfections was relatively small. The interpretation and extrapolation of these results should be conducted with caution. A multicenter prospective study with a larger sample is needed. Third, the epidemiology level of adenovirus coinfection with other pathogens in the general population was unclear in this research area.

The world is at a new stage of the COVID-19 pandemic. With non-pharmaceutical interventions (NPIs) having been released, HAdV and HCoVs, including SARS-CoV-2, are circulating endemically in human populations. The early signs and symptoms of infection with adenovirus and HCoV-229E are similar to those of COVID-19 (27). In the future, physicians and public health doctors should be alert to the possibility of HAdV coinfection during the COVID-19 pandemic.

## Data availability statement

The original contributions presented in the study are included in the Supplementary material for this article. All sequences from mono-infections with adenovirus and the HCoV 229E, and coinfections with adenovirus and the HCoV-229E were submitted to the NIH genetic sequence database (GenBank, https://www.ncbi.nlm.nih.gov/genbank/). The number of the sequence GenBank are seen in Supplementary Table 1. Further inquiries can be directed to the corresponding author/s.

## Ethics statement

The studies involving human participants were reviewed and approved by the study was approved by the Medical Ethics Committee of the Zhejiang Province Center for Disease Control and Prevention (No. 2019013). Written consent was obtained from all patients or their family. Written informed consent to participate in this study was provided by the participants' legal guardian/next of kin.

## Author contributions

SL drafted the first version of the manuscript and obtained funding for the study. AZ, JP, LY, WS, and HW collected clinical data for the study. HZ and HL verified and analyzed data for the study. LW, SQ, ZY, JC, and EC conducted the investigation. YC analyzed all samples. All of the authors contributed data to the study and participated in data interpretation and critical review of the manuscript. All of the authors approved the final manuscript for submission.

## Funding

This work was supported by the Zhejiang Provincial Program for The Cultivation of High-Level Innovative Health Talents.

## Conflict of interest

The authors declare that the research was conducted in the absence of any commercial or financial relationships that could be construed as a potential conflict of interest.

## Publisher's note

All claims expressed in this article are solely those of the authors and do not necessarily represent those of their affiliated organizations, or those of the publisher, the editors and the reviewers. Any product that may be evaluated in this article, or claim that may be made by its manufacturer, is not guaranteed or endorsed by the publisher.

## References

[1] Charman M Herrmann C Weitzman MD. Viral and cellular interactions during adenovirus DNA replication. FEBS Lett. (2019) 593:3531–50. 10.1002/1873-3468.13695 31764999 PMC6928415

[2] Hage E Dhingra A Liebert UG Bergs S Ganzenmueller T Heim A. Three novel, multiple recombinant types of species of human mastadenovirus D(HAdV-D 73, 74, and 75) isolated from diarrhoeal faeces of immunocompromised patients. J Gen Virol. (2017) 98:3037–45. 10.1099/jgv.0.000968 29095687

[3] Li J Lu X Sun Y Lin C Li F Yang Y. swimming pool-associated outbreak of pharyngoconjunctival fever caused by human adenovirus type 4 in Beijing, China. Int J Infect Dis. (2018) 75:89–91. 10.1016/j.ijid.2018.08.009 30144556 PMC6198331

[4] Vassilara F Spyridaki A Pothitos G Deliveliotou A Papadopoulos A. A rare case of human coronavirus 229E associated with acute respiratory distress syndrome in a healthy adult. Case Rep Infect Dis. (2018) 2018:6796839. 10.1155/2018/6796839 29850307 PMC5925015

[5] Kajon AE Lamson DM Bair CR Lu X Landry ML Menegus M. Adenovirus type 4 respiratory infections among civilian adults, northeastern United States, 2011-2015(1). Emerg Infect Dis. (2018) 24:201–9. 10.3201/eid2402.171407 29350143 PMC5782899

[6] Lynch BL Dean J Brady D De Gascun C. Adenovirus type 4 respiratory infections among civilian adults, northeastern United States, 2011-2015. Emerg Infect Dis. (2018) 24:1392–3. 10.3201/eid2407.180137 29912699 PMC6038765

[7] Binder AM Biggs HM Haynes AK Chommanard C Lu X Erdman DD. Human adenovirus surveillance—United States, 2003–2016. MMWR Morb Mortal Wkly Rep. (2017) 66:1039–42. 10.15585/mmwr.mm6639a2 28981484 PMC5720882

[8] CDC. Adenovirus. (2019). Available online at: https://www.cdc.gov/adenovirus/adenovirus-factsheet-508.pdf (accessed August 28, 2019)

[9] Shieh WJ. Human adenovirus infections in pediatric population—an update on clinico-pathologic correlation. Biomed J. (2022) 45:38–49. 10.1016/j.bj.2021.08.009 34506970 PMC9133246

[10] Gao J Xu L Xu B Xie Z Shen K. Human adenovirus Coinfection aggravates the severity of *Mycoplasma* pneumoniae pneumonia in children. BMC Infect Dis. (2020) 20:420. 10.1186/s12879-020-05152-x 32546135 PMC7296888

[11] Ghebremedhin B. Human adenovirus: viral pathogen with increasing importance. Eur J Microbiol Immunol. (2014) 4:26–33. 10.1556/EuJMI.4.2014.1.2 24678403 PMC3955829

[12] Xu Z Shi L Wang Y Zhang J Huang L Zhang C. Pathological findings of COVID-19 associated with acute respiratory distress syndrome. Lancet Respir Med. (2020) 8:420–2. 10.1016/S2213-2600(20)30076-X 32085846 PMC7164771

[13] Mulabbi EN Tweyongyere R Byarugaba DK. The history of the emergence and transmission of human coronaviruses. Onderstepoort J Vet Res. (2021) 88:e1–8. 10.4102/ojvr.v88i1.1872 33567843 PMC7876959

[14] Kesheh MM Hosseini P Soltani S Zandi M. An overview on the seven pathogenic human coronaviruses. Rev Med Virol. (2022) 32:e2282. 10.1002/rmv.2282 34339073

[15] Aleebrahim-Dehkordi E Soveyzi F Deravi N Rabbani Z Saghazadeh A Rezaei N. Human coronaviruses SARS-CoV, MERS-CoV, and SARS-CoV-2 in children. J Pediatr Nurs. (2021) 56:70–9. 10.1016/j.pedn.2020.10.020 33186866 PMC7580518

[16] Ma Z Li P Ji Y Ikram A Pan Q. Cross-reactivity towards SARS-CoV-2: the potential role of low-pathogenic human coronaviruses. Lancet Microbe. (2020) 1:e151. 10.1016/S2666-5247(20)30098-7 33521716 PMC7836609

[17] CDC. Human Coronavirus Types. (2020). Available online a: https://www.cdc.gov/coronavirus/types.html (accessed February 15, 2020).

[18] Probst V Spieker AJ Stopczynski T Stewart LS Haddadin Z Selvarangan R. Clinical presentation and severity of adenovirus detection alone vs adenovirus co-detection with other respiratory viruses in US children with acute respiratory illness from 2016 to 2018. J Pediatric Infect Dis Soc. (2022) 11:430–9. 10.1093/jpids/piac066 35849119 PMC13396784

[19] Feng L Li Z Zhao S Nair H Lai S Xu W. Viral etiologies of hospitalized acute lower respiratory infection patients in China, 2009–2013. PLoS ONE. (2014) 9:e99419. 10.1371/journal.pone.0099419 24945280 PMC4063718

[20] Huang Y Wang C Ma F Guo Q Yao L Chen A. Human adenoviruses in paediatric patients with respiratory tract infections in Beijing, China. Virol J. (2021) 18:191. 10.1186/s12985-021-01661-6 34556127 PMC8460180

[21] Ravi RK Walton K Khosroheidari M. MiSeq: a next generation sequencing platform for genomic analysis. Methods Mol Biol. (2018) 1706:223–32. 10.1007/978-1-4939-7471-9_12 29423801

[22] Biere B Oh DY Wolff T Durrwald R. Surveillance of endemic human Coronaviruses in Germany, 2019/2020. Lancet Reg Health Eur. (2021) 11:1–4. 10.1016/j.lanepe.2021.100262 34751265 PMC8566015

[23] Otieno GP Murunga N Agoti CN Gallagher KE Awori JO Nokes DJ. Surveillance of endemic human coronaviruses(HCoV-NL63, OC43 and 229E) associated with childhood pneumonia in Kilifi, Kenya. Wellcome Open Res. (2020) 5:150. 10.12688/wellcomeopenres.16037.2 32995556 PMC7512035

[24] Shi Y Shi J Sun L Tan Y Wang G Guo F. Insight into vaccine development for Alpha-coronaviruses based on structural and immunological analyses of spike proteins. J Virol. (2021). 10.1128/JVI.02284-20 33414160 PMC8092709

[25] Liu S Cai J Li Y Ying L Li H. Outbreak of acute respiratory disease caused by human adenovirus type 7 and human coronavirus-229E in Zhejiang Province, China. J Med Virol. (2022) 28. 10.1002/jmv.28101 36031726

[26] Lessler J Reich NG Brookmeyer R Perl TM Nelson KE Cummings DA. Incubation periods of acute respiratory viral infections: a systematic review. Lancet Infect Dis. (2009) 9:291–300. 10.1016/S1473-3099(09)70069-6 19393959 PMC4327893

[27] Imran M Yasmeen R. SARS-CoV2 Outbreak: Emergence, transmission and clinical features of human coronaviruses. J Ayub Med Coll Abbottabad. (2020) 32:S710–3. 33754539

[28] Li SW Lin CW. Human coronaviruses: Clinical features and phylogenetic analysis. Biomedicine. (2013) 3:43–50. 10.1016/j.biomed.2012.12.007 32289002 PMC7103958

[29] Varghese L Zachariah P Vargas C LaRussa P Demmer RT Furuya YE. Epidemiology and clinical features of human coronaviruses in the pediatric population. J Pediatric Infect Dis Soc. (2018) 7:151–8. 10.1093/jpids/pix027 28482105 PMC5954244

[30] Lai S Ruktanonchai NW Zhou L Prosper O Luo W Floyd JR. Effect of non-pharmaceutical interventions to contain COVID-19 in China. Nature. (2020) 585:410–3. 10.1038/s41586-020-2293-x 32365354 PMC7116778

[31] Shou MH Wang ZX Lou WQ. Effect evaluation of non-pharmaceutical interventions taken in China to contain the COVID-19 epidemic based on the susceptible-exposed-infected-recovered model. Technol Forecast Soc Chang. (2021) 171:120987. 10.1016/j.techfore.2021.120987 34176979 PMC8220917

[32] Wei J Wu S Jin X Zhang J Pan S. Association of Mycoplasma pneumoniae coinfection with adenovirus pneumonia severity in children. Allergol Immunopathol. (2022) 50:31–6. 10.15586/aei.v50i1.476 34873894

[33] Wu PQ Zeng SQ Yin GQ Huang JJ Xie ZW Lu G. Clinical manifestations and risk factors of adenovirus respiratory infection in hospitalized children in Guangzhou, China during the 2011-2014 period. Medicine. (2020) 99:e18584. 10.1097/MD.0000000000018584 31977849 PMC7004600

[34] Chen LL Cheng YG Chen ZM Li SX Li XJ Wang YS. Mixed infections in children with *Mycoplasma* pneumoniae pneumonia. Zhonghua Er Ke Za Zhi. (2012) 50:211–5. 22801206

[35] Cai R Mao N Dai J Xiang X Xu J Ma Y. Correction: genetic variability of human adenovirus type 7 circulating in mainland China. PLoS ONE. (2020) 15:e0234681. 10.1371/journal.pone.0234681 32516329 PMC7282639

[36] Cai R Mao N Dai J Xiang X Xu J Ma Y. Genetic variability of human adenovirus type 7 circulating in mainland China. PLoS ONE. (2020) 15:e0232092. 10.1371/journal.pone.0232092 32352995 PMC7192419

[37] Huong Ple T Hien PT Lan NT Binh TQ Tuan DM Anh DD. First report on prevalence and risk factors of severe atypical pneumonia in Vietnamese children aged 1-15 years. BMC Public Health. (2014) 14:1304. 10.1186/1471-2458-14-1304 25524126 PMC4300840

[38] Motta JC Gomez CC. Adenovirus and novel coronavirus(SARS-Cov2) coinfection: a case report. IDCases. (2020) 22:e00936. 10.1016/j.idcr.2020.e00936 32864341 PMC7443165

[39] Baker JM Buchfellner M Britt W Sanchez V Potter JL Ingram LA. Acute hepatitis and adenovirus infection among children—Alabama, October 2021–February 2022. MMWR Morb Mortal Wkly Rep. (2022) 71:638–40. 10.15585/mmwr.mm7118e1 35511732 PMC9098244

[40] Baker JM Buchfellner M Britt W Sanchez V Potter JL Ingram LA. Acute hepatitis and adenovirus infection among children—Alabama, October 2021–February 2022. Am J Transpl. (2022) 22:1919–21. 10.1111/ajt.16665 35789534

[41] Gutierrez Sanchez LH Shiau H Baker JM Saaybi S Buchfellner M Britt W. A case series of children with acute hepatitis and human adenovirus infection. N Engl J Med. (2022) 387:620–30. 10.1056/NEJMoa2206294 35830653 PMC9808750

[42] Hakim MS. The recent outbreak of acute and severe hepatitis of unknown etiology in children: a possible role of human adenovirus infection? J Med Virol. (2022) 94:4065–8. 10.1002/jmv.27856 35577525

[43] Paraskevis D Papathedoridis G Sypsa V Sfikakis P Tsiodras S Zaoutis T. Alpha proposed etiology for an aberrant response to enteric adenovirus infection in previously SARS-CoV-2-infected children with acute hepatitis. J Pediatric Infect Dis Soc. (2022). 10.1093/jpids/piac053 35674694 PMC9384245

[44] Shan S Jia JD. The relationship between adenovirus infection and severe acute hepatitis of unknown etiology. Zhonghua Gan Zang Bing Za Zhi. (2022) 30:470–2. 10.3760/cma.j.cn501113-20220429 35764537 PMC12769886

[45] van Tol MJ Kroes AC Schinkel J Dinkelaar W Claas EC. Jol-van der Zijde CM. Adenovirus infection in paediatric stem cell transplant recipients: increased risk in young children with a delayed immune recovery. Bone Marrow Transpl. (2005) 36:39–50. 10.1038/sj.bmt.1705003 15908981

