# Supplementary material for: The clinical and virological features of two children's coinfections with human adenovirus type 7 and human coronavirus-229E virus
Source: Front Public Health. 2022 Nov 15;10:1048108. doi: 10.3389/fpubh.2022.1048108 (PMC9706225; doi:10.3389/fpubh.2022.1048108)
Supplement: Supplementary file 1 [file Data_Sheet_1.docx]

**Supplementary Table 1: GenBank accession numbers of the hexon, the fiber, and the penton of the HAdV, and S1 gene of the HCoV 229E isolated from the mono- or co-infections in JinYun Countryside, Lishui City, Zhejiang Province, China during July and August 2019**

| Groups | Case type | Sampling date | Type of samples | GenBank accession numbers | | | |
| --- | --- | --- | --- | --- | --- | --- | --- |
|  |  |  |  | HCoV 229E S1 gene | HAdV Penton | HAdV Hexon | HAdV Fiber |
| Co-infections | Co-infection 1 | 2-8-2019 | Pharyngeal swab | OP131705 | OP131668 | OP131646 | OP131689 |
|  | Co-infection 2 | 2-8-2019 | Pharyngeal swab | OP131706 | OP131673 | OP131651 | OP131694 |
| Mono-Adenovirus | Mono-infection 1 | 2-8-2019 | Pharyngeal swab | / | OP131663 | OP131641 | OP131684 |
|  | Mono-infection 2 | 2-8-2019 | Pharyngeal swab | / | OP131664 | OP131642 | OP131685 |
|  | Mono-infection 3 | 2-8-2019 | Pharyngeal swab | / | OP131665 | OP131643 | OP131686 |
|  | Mono-infection 4 | 2-8-2019 | Pharyngeal swab | / | OP131666 | OP131644 | OP131687 |
|  | Mono-infection 5 | 2-8-2019 | Pharyngeal swab | / | OP131667 | OP131645 | OP131688 |
|  | Mono-infection 6 | 2-8-2019 | Pharyngeal swab | / | OP131669 | OP131647 | OP131690 |
|  | Mono-infection 7 | 2-8-2019 | Pharyngeal swab | / | OP131670 | OP131648 | OP131691 |
|  | Mono-infection 8 | 2-8-2019 | Pharyngeal swab | / | OP131671 | OP131649 | OP131692 |
|  | Mono-infection 9 | 2-8-2019 | Pharyngeal swab | / | OP131672 | OP131650 | OP131693 |
|  | Mono-infection 10 | 2-8-2019 | Pharyngeal swab | / | OP131674 | OP131652 | OP131695 |
|  | Mono-infection 11 | 2-8-2019 | Pharyngeal swab | / | OP131675 | OP131653 | OP131696 |
|  | Mono-infection 12 | 2-8-2019 | Pharyngeal swab | / | OP131676 | OP131654 | OP131697 |
|  | Mono-infection 13 | 2-8-2019 | Pharyngeal swab | / | OP131677 | OP131655 | OP131698 |
|  | Mono-infection 14 | 2-8-2019 | Pharyngeal swab | / | OP131678 | OP131656 | OP131699 |
|  | Mono-infection 15 | 2-8-2019 | Pharyngeal swab | / | OP131679 | OP131657 | OP131700 |
|  | Mono-infection 16 | 2-8-2019 | Conjunctival swab | / | OP131680 | OP131658 | OP131701 |
|  | Mono-infection 17 | 3-8-2019 | Pharyngeal swab | / | OP131682 | OP131660 | OP131703 |
| Mono- HCoV 229E | Mono-infection 1 | 2-8-2019 | Pharyngeal swab | OP131708 | / | / | / |
|  | Mono-infection 2 | 2-8-2019 | Pharyngeal swab | OP131707 | / | / | / |

Notes:/ means No available


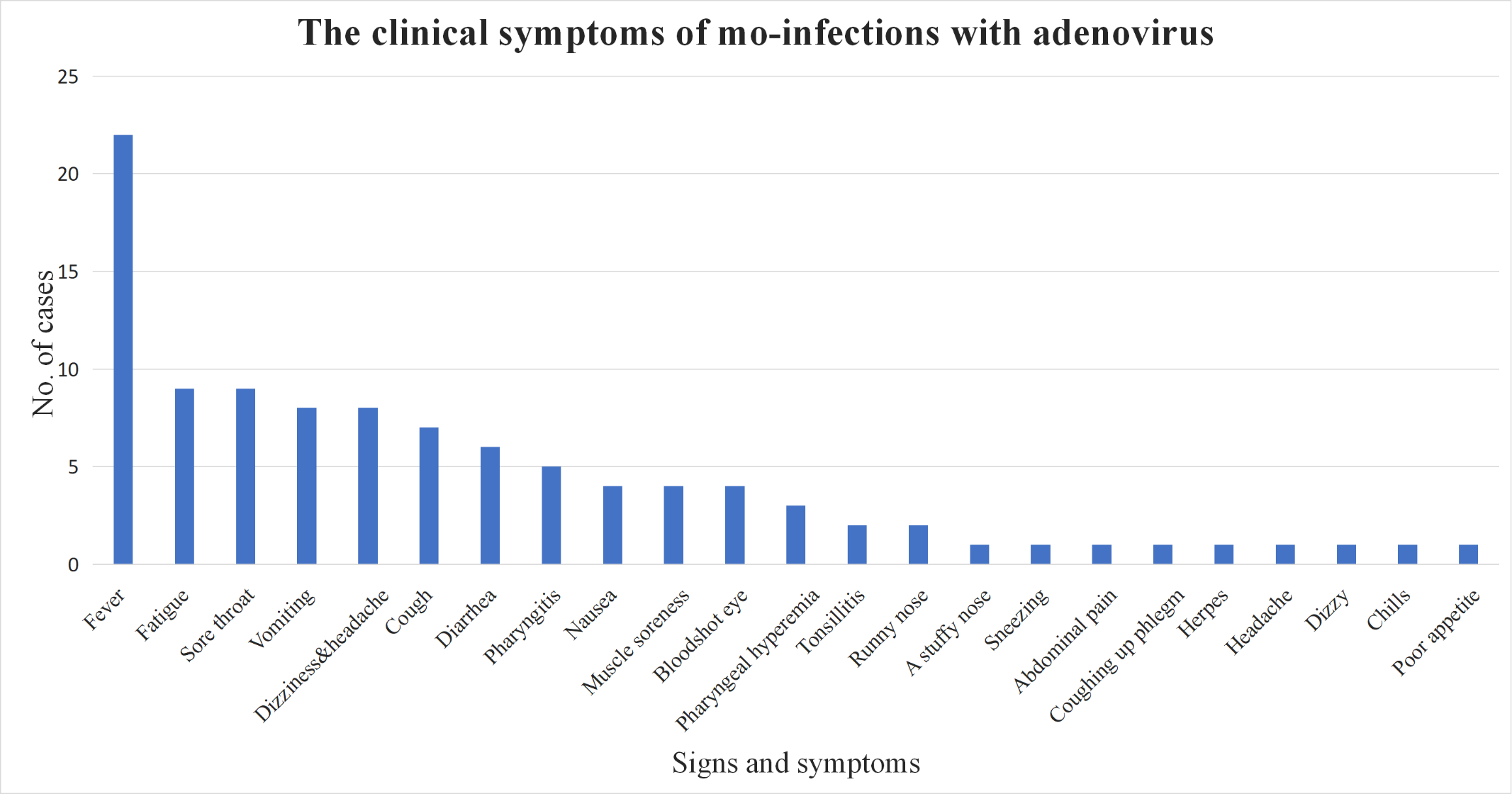


**Supplementary Figure 1: The clinical symptoms and signs of the 21 mono-adenovirus patients, Zhejiang Province, China during July and August 2019**
